# Computation of Electronic Bound States in Anionic Clusters as Precursors to Solvated Electrons

**DOI:** 10.1021/acs.jctc.6c00731

**Published:** 2026-06-16

**Authors:** Xiangfei Wang

**Affiliations:** Institute of Chemistry and Biochemistry, 9166 Freie Universität Berlin, Arnimallee 22, 14195 Berlin, Germany

## Abstract

Solvated electrons are highly reactive species that drive a wide range of redox reactions. In this study, we compute electronic bound states in anionic molecular clusters as model systems for solvated electrons. Our method accounts for solvent polarization induced by the excess electron and electron correlation through a hybrid two-step scheme. Across a range of electron-binding strengths, the resulting bound-state energies reproduce CCSD­(T) vertical detachment energies, with fitted slopes deviating from unity by less than 5%. The absolute errors are also at least 30 meV smaller than those from commonly used MP2 and range-separated ΔSCF calculations. The corresponding bound-state wave functions reproduce the spatial distribution of the excess electron. For the tested structures, the computational cost scales quartically. Overall, our approach provides an efficient framework for describing the ground state of the excess electron within a one-orbital picture.

## Graphical abstract

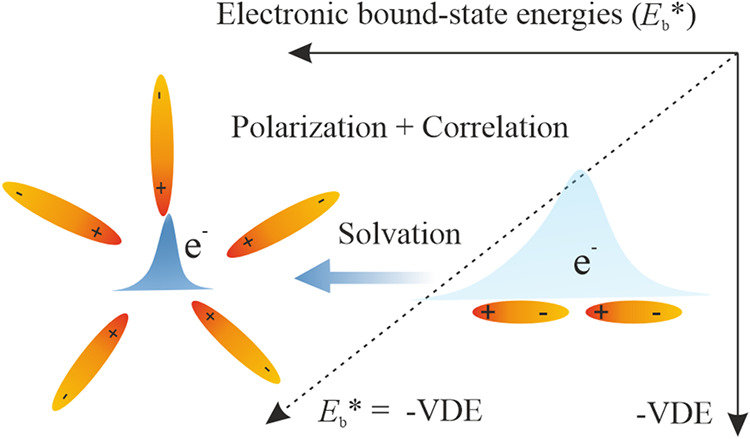

## 1 Introduction

The solvated electron, identified by Hart and Boag,^1^ is a highly reactive reducing agent that drives a wide range of radiation-chemical and biological processes,^2^ such as photocatalysis,^3^ redox reactions,^4^ and radiation-induced DNA damage.^5^ In addition to water, it exists in various solvents, including ammonia,^6^ amines (e.g., methylamine and ethylamine),^7,8^ methanol,^9^ and ethanol.^10^ Substantial progress has been made toward understanding the structure and dynamics of solvated electrons.^11,12^ Nevertheless, open questions remain regarding how their binding, localization, and dynamics depend on the solvent environment, continuing to motivate spectroscopic and theoretical studies.^13^

The hydrated electron in liquid water is among the most widely studied solvated-electron systems and forms on ultrafast time scales. After being released, the excess electron rapidly propagates along the hydrogen-bond network.^14,15^ It is then trapped and becomes localized in a cavity formed by solvent reorientation on the subpicosecond scale.^16–18^ The fully solvated electron exhibits a broad absorption band near 700 nm (1.70 eV),^1^ which is commonly assigned to an s → p transition. The bound state is characterized by a vertical detachment energy (VDE) of ∼3.70–3.80 eV.^19,20^ In comparison, liquid ammonia provides another classic solvent environment, with an s → p transition near 1500 nm (0.83 eV)^21^ and a substantially smaller VDE of ∼2.00 eV.^22^

To understand how solvents stabilize an excess electron, simulations provide molecular-level insight from both periodic models and finite clusters. Periodic MP2 (Møller–Plesset second-order perturbation theory) calculations for a 47-water cell show that elongated hydrogen bonds act as transient electron traps, followed by cavity formation on subpicosecond time scales.^23^ Longer-time trajectory simulations based on neural-network potentials trained on MP2 data suggest that full stabilization can require up to ∼1 ps.^24^ In addition, finite water clusters allow electron localization and binding to be tuned systematically. For (H_2_O)_*n*_^–^ (*n* = 4–6), the excess electron partially occupies O–H antibonding orbitals, which weakens O–H bonds and contributes to a red-shift in the IR spectrum.^25^ For *n* = 20–200, effective-potential simulations reveal a strong relationship between the optical absorption maximum and the electron size (radius of gyration, RG).^26^

Simulating solvated electrons is computationally demanding because the cost of high-level electronic-structure methods grows rapidly with system size. As a result, large water clusters are often treated using effective potential or mixed quantum/classical approaches,^26^ whereas *ab initio* benchmarks are typically restricted to smaller clusters and representative models. This limitation motivates benchmarking electronic-structure methods that balance accuracy and efficiency by analyzing VDEs and the degree of excess-electron localization.

The most widely used reference methods for solvated-electron benchmarks are wave function approaches, most notably CCSD­(T) (coupled-cluster singles and doubles with perturbative triples) and MP2. CCSD­(T), often regarded as a gold standard in quantum chemistry,^27^ provides benchmark VDEs for small anionic water clusters (*n* ≤ 6).^28,29^ However, CCSD­(T) is computationally demanding and typically scales as *O*(*N* ^7^) with the number of basis functions *N*. MP2 offers a lower-cost alternative with a typical scaling of *O*(*N* ^5^). Herbert et al.^30^ showed that MP2 reproduces CCSD­(T) VDEs for strongly bound cluster anions with an average deviation of ∼7%, enabling studies up to *n* = 24. In parallel, more computationally efficient MP2 implementations have also been developed, including local-correlation approaches^31^ and tensor-hypercontraction-based formulations, for which quartic-scaling algorithms have been reported.^32^

Kohn–Sham density functional theory (KS-DFT) offers a more scalable approach (∼*O*(*N* ^3^)) for describing excess-electron binding and localization. The VDE can be evaluated using ΔSCF (self-consistent field) as the energy difference between the anionic and neutral clusters at the same geometry. Its accuracy depends critically on the choice of exchange–correlation functional and can be improved through careful tuning of range-separated functionals,^33^ for example, by enforcing the ionization-potential condition. Yagi et al.^34^ showed that tuning the long-range exchange in a range-separated functional yields VDEs with a mean absolute error (MAE) of 35.2 meV relative to CCSD­(T) for small water clusters with *n* = 2–6. Nevertheless, loosely bound excess electrons in small clusters are stabilized by long-range charge–multipole interactions and therefore exhibit highly diffuse densities.^35^ This diffuse character makes self-interaction error and incorrect asymptotic behavior of common density functional approximations (DFAs) particularly problematic.^36,37^

Accurate long-range exchange is essential for describing anionic molecular clusters. In this work, we adopt a frozen-target static-exchange (SE) picture from the low-energy electron scattering process.^38–40^ Exchange is represented by the exact exchange evaluated from the valence KS orbitals and included as an effective one-electron term acting on the excess electron. Our method also accounts for (i) polarization of the valence-electron density by the excess electron and (ii) correlation between the excess electron and the valence electrons. Within this framework, the excess electron is described in an effective single-orbital picture: its orbital energy reproduces the VDE, and its wave function represents the spatial distribution of the excess electron.

In this study, we assess the proposed computational scheme in terms of bound-state energies, spatial distributions of wave functions, and computational cost. First, we compare the computed bound-state energies with reference VDEs of the anionic clusters. We also compare the bound-state energies with VDEs obtained from the well-established range-separated ΔSCF and MP2 calculations to assess whether the proposed scheme improves the accuracy. The robustness of the bound-state energies is further examined by benchmarking different DFT starting points used to construct the effective potential. Second, we validate the bound-state wave functions by comparing their spatial distributions with the excess-electron densities. Third, we analyze the computational time and its scaling with the number of basis functions to evaluate the feasibility of extending the method to larger structures. To assess the generality of the method, we perform these analyses for multiple solvents, including water, ammonia, and methanol.

## 2 Theory and Methods

### 2.1 Exact Exchange in the Effective Potential

In the SE approximation, the target molecule is kept frozen in a reference electronic state, while the scattering electron moves in the electrostatic field and exchange potential generated by the occupied target orbitals.^41–43^ In the present work, we adopt SE to construct an effective one-electron Hamiltonian for the excess electron. This frozen-solvent description is supplemented by a separate polarization step, in which the valence molecular orbitals are allowed to relax in the presence of excess electron. Compared with a fully self-consistent all-electron treatment, SE remains an approximate description. The solvent electron density is allowed to polarize in response to the excess electron, but not to undergo the full redistribution of the electron density, which is more common in chemical reactions.

Specifically, the exchange operator *V̂*_*x*_^EXX^[{ϕ_*i*_}] is constructed from the occupied KS orbitals {ϕ_*i*_} and included in the effective potential acting on the excess electron, as shown below:

1 $${Ĥ}_{\mathrm{SE}}=\mathrm{T̂}+{\mathrm{V̂}}_{\mathrm{ext}}+{\mathrm{V̂}}_{H}\left[\rho_{0}\right]+{\mathrm{V̂}}_{x}^{\mathrm{EXX}}\left[\{\phi_{i}\}\right]$$

2 $$\mathrm{T̂}=-\frac{1}{2}\nabla^{2}$$

where *V̂*_ext_ is the electrostatic potential due to the nuclei and *V̂*_H_[ρ_0_] is the Hartree (Coulomb) potential generated by the frozen *N*_e_-electron density ρ_0_. The operator *T̂* denotes the one-electron kinetic-energy term. The index *i* labels the occupied KS orbitals {ϕ_*i*_}, which form a closed-shell configuration of the valence electrons. The reference density ρ_0_ is constructed from the occupied orbitals.

Together, *Ĥ*_SE_ in eq 1 defines an effective one-electron Hamiltonian for the excess electron. Solving *Ĥ*_SE_ yields a trial wave function Ψ_sol,0_ and the corresponding energy *E*_sol,0_. At this stage, Ψ_sol,0_ and *E*_sol,0_ provide only an estimate of the excess-electron distribution and binding energy. This is because *Ĥ*_SE_ does not include a full description of solvent–electron interactions.

In addition, we solve *Ĥ*_SE_ in the subspace spanned by the virtual (unoccupied) KS orbitals {ϕ_*a*_}, where *a* labels the virtual orbitals. We restrict the solution to this virtual subspace because the occupied KS orbitals {ϕ_*i*_} are already doubly occupied in the closed-shell configuration. Accommodation of an additional electron is forbidden by the Pauli exclusion principle. The resulting equations are

3 $${\mathrm{P̂}}_{\mathrm{virt}}{Ĥ}_{\mathrm{SE}}{\mathrm{P̂}}_{\mathrm{virt}}\Psi_{\mathrm{sol},0}=E_{\mathrm{sol},0}\Psi_{\mathrm{sol},0}$$

4 $${\mathrm{P̂}}_{\mathrm{virt}}=\sum_{a}\left|\phi_{a}\right\rangle \left\langle \phi_{a}\right|$$

where *P̂*_virt_ denotes the projection operator onto the virtual subspace spanned by the KS virtual orbitals {ϕ_*a*_}. The subscript 0 on *E*_sol,0_ and Ψ_sol,0_ indicates the lowest eigenvalue and the corresponding eigenfunction, respectively. In the following sections, we refine the effective Hamiltonian by allowing solvent-density polarization and by incorporating correlation between the excess electron and the valence electrons.

### 2.2 Electrostatic Polarization of the Solvent

The excess electron perturbs the solvent electronic structure through its Coulomb potential. As a result, the solvent electron density becomes polarized, and the orbitals change accordingly. To account for this polarization, we include the excess-electron potential in the solvent KS-DFT Hamiltonian and update it iteratively as follows.

5 $$\left[\mathrm{T̂}+{\mathrm{V̂}}_{\mathrm{ext}}+{\mathrm{V̂}}_{H}\left[\rho_{k}\right]+{\mathrm{V̂}}_{\mathrm{xc}}\left[\rho_{k},\phi_{i,k}\right]+{\mathrm{V̂}}_{\mathrm{sol}}\left[\Psi_{\mathrm{sol},0}\right]\right]\phi_{i,k+1}=\epsilon_{i,k+1}\phi_{i,k+1}$$

where the index *k* labels the iterations in the self-consistent procedure. The operator *V̂*_xc_[ρ_*k*_,{ϕ_*i*,*k*_}] denotes the exchange–correlation potential of the chosen KS-DFT functional. For modern hybrid^44–46^ and range-separated hybrid functionals,^47–49^ this operator depends on both the density ρ_*k*_ and the occupied orbitals {ϕ_*i*,*k*_}. The operator *V̂*_sol_[Ψ_sol,0_] denotes the Coulomb potential of the excess electron evaluated from the trial wave function Ψ_sol,0_.

Therefore, eq 5 can be solved efficiently using a standard KS-SCF procedure. For a fixed trial wave function Ψ_sol,0_, the SCF cycle is iterated until the solvent orbitals {ϕ_*i*,*k*_} and electron density ρ_*k*_ converge to {ϕ_i_^*^} and ρ*, respectively. Because the SCF problem is solved in the presence of excess-electron potential, {ϕ_i_^*^} and ρ* represent the polarized solvent orbitals and the corresponding induced electron density.

To include the feedback of the polarized solvent on the excess electron, we update *Ĥ*_SE_ by inserting {ϕ_i_^*^}, {ϕ_a_^*^}, and ρ* into eq 1. We then solve the updated *Ĥ*_SE_ in eq 3 to obtain a trial wave function and energy, Ψ_sol,0_^p^ and *E*_sol,0_^p^. Here, p labels the outer iteration used to update the excess-electron wave function and energy. We repeat the inner solvent-SCF cycle and the outer excess-electron step until Ψ_sol,0_^p^ and *E*_sol,0_^p^ converge. The converged trial wave function and energy are denoted by Ψ_sol,0_^*^ and *E*_sol,0_^*^.

### 2.3 Electron Correlation

The correlation energy can be essential for achieving chemical accuracy and reliable theoretical interpretations,^50^ but it accounts for only a small fraction of the total electronic energy. Therefore, it is common to start from a converged mean-field reference and recover correlation effects using perturbation theory.^51,52^ Following a similar strategy, we treat the correlation between the excess electron and the valence electrons as a final (one-shot) correction after the electrostatic polarization cycle has converged to *E*_sol,0_^*^ and Ψ_sol,0_^*^.

We account for correlation effects within the *GW* approximation.^53–55^ Specifically, this study employs the non-self-consistent *G*_0_ *W*_0_ approach. The central quantity is the self-energy Σ, which depends on the screened Coulomb interaction *W*. The screening is governed by the solvent dielectric response and is described by the frequency-dependent dielectric function ϵ­(ω). In our implementation, ϵ­(ω), and thus *W*, is obtained from the independent-particle susceptibility χ_0_(ω) constructed from KS orbitals and their eigenvalues. This mean-field treatment of dielectric screening is commonly referred to as the random-phase approximation (RPA).^56,57^

6 $$\mathrm{\Sigma ̂}\left(\omega \right)=\frac{i}{2\pi }\int_{-\infty }^{+\infty }d\omega^{\prime }\;Ĝ\left(\omega +\omega^{\prime }\right)Ŵ\left(\omega^{\prime }\right)$$

7 $$Ŵ\left(\omega \right)={\mathrm{\varepsilon ̂}}^{-1}\left(\omega \right)\mathrm{v̂}$$

where *Ĝ* is Green’s function constructed from the KS orbitals and energies and *v̂* is the bare Coulomb kernel. For the self-energy of the excess electron, we evaluate Σ̂(ω) only within the virtual-orbital subspace, which reduces the computational cost. In addition, eq 6 requires integration along the real frequency axis. Because this contour passes close to the poles of *Ĝ* and *Ŵ*, the numerical integration becomes challenging. Therefore, we follow Zhu et al.^58^ by performing the integration along the imaginary axis and then obtaining the corresponding real-axis values via extrapolation.

To refine the bound-state energy and wave function, we incorporate the self-energy correction Σ̂(ω) into eq 1. This yields an energy-dependent, correlation-corrected effective Hamiltonian *Ĥ*_full_(ω), given by

8 $${Ĥ}_{\mathrm{full}}\left(\omega \right)=\mathrm{T̂}+{\mathrm{V̂}}_{\mathrm{ext}}+{\mathrm{V̂}}_{H}\left[\rho^{*}\right]+{\mathrm{V̂}}_{x}^{\mathrm{EXX}}\left[\{\phi_{i}^{*}\}\right]+\mathrm{\Sigma ̂}\left(\omega \right)$$

Here, ω denotes the bound-state energy of the excess electron and is determined self-consistently. We use ω^0^ = *E*_sol,0_^*^ (eq 3) as the initial guess and then solve the following equations iteratively:

9 $${\mathrm{P̂}}_{\mathrm{virt}}^{*}{Ĥ}_{\mathrm{full}}\left(\omega^{m}\right){\mathrm{P̂}}_{\mathrm{virt}}^{*}\psi_{b}^{m+1}=\omega^{m+1}\psi_{b}^{m+1}$$

10 $${\mathrm{P̂}}_{\mathrm{virt}}^{*}=\sum_{a}\left|\phi_{a}^{*}\right\rangle \left\langle \phi_{a}^{*}\right|$$

In the above equation, ψ_b_^*m*^ and ω^*m*^ denote the correlation-inclusive wave function and eigenvalue of the excess electron at the *m*th iteration. Upon convergence, these quantities are written as ψ_b_^*^ and ω*. In the following, we define the converged eigenvalue ω* as the electronic bound-state energy, denoted by *E*_b_^*^. Because the non-self-consistent *G*_0_ *W*_0_ method depends on the reference orbitals, we further use *E*_b_^*,XC^ and ψ_b_^*,XC^ to denote the bound-state energy and wave function obtained from the proposed scheme. Here, XC denotes the starting exchange–correlation functional used to generate the KS orbitals and energies.

## 3 Computational Details

All calculations in this work employ the 6-31­(1+,3+)­G** basis set. This basis set is derived from 6 to 31­(1+,3+)­G*, which has been tested in previous CCSD­(T) and MP2 benchmark studies of anionic water clusters.^30^ Compared with 6-31­(1+,3+)­G*, 6-31­(1+,3+)­G** includes an additional polarization function on hydrogen. These extra *p* functions provide additional angular flexibility for describing the polarization response of the cluster and can thereby improve the description of the excess electron distribution.

The present electronic bound-state calculations depend directly on the underlying exchange–correlation functional and its range-separation implementation. Therefore, all DFT-related calculations, including the ΔSCF calculations, were carried out using the PySCF package^59^ (v2.12.1) to ensure consistent functional conventions, range-separation, and numerical settings. Only the wave function methods, including MP2 and CCSD­(T), were carried out with the TURBOMOLE (v7.6.1) program suite.^60^

We use VDEs evaluated at the CCSD­(T) level rather than MP2-level VDEs as the reference throughout this study. The deviations of the present electronic bound state energies from the CCSD­(T) VDEs are comparable to the typical differences between MP2 and CCSD­(T) VDEs. Therefore, using MP2VDEs as the reference would provide a less stringent benchmark and would be less able to resolve small differences in the accuracy. The MP2 and CCSD­(T) VDE data are provided in the Supporting Information.

The cluster geometries are obtained by optimizing the corresponding anionic structures at the MP2 level. For (H_2_O)_*n*_^–^ and (NH_3_)_*n*_^–^, the initial geometries are taken from Kim et al.^28^ and Baranyi et al.,^61^ respectively. Methanol cluster anions are generated similarly by constructing hydrogen-bonded initial structures and then optimizing the anionic clusters at the MP2 level.

For each cluster, the VDE is defined as the total-energy difference between the neutral (*E*_neu_) and anionic (*E*_an_^–^) clusters evaluated at the anion geometry (**R**_an_),

11 $$E_{\mathrm{VDE}}=E_{\mathrm{neu}}\left(R_{\mathrm{an}}\right)-E_{\mathrm{an}}^{-}\left(R_{\mathrm{an}}\right)$$

We evaluate the VDE at several levels of theory, including CCSD­(T) (*E*_VDE_^CCSD(T)^), MP2 (*E*_VDE_^MP2^), and ΔSCF calculations with different exchange–correlation functionals (*E*_VDE_^XC^).

To characterize the spatial distribution of the excess electron, we construct spin-resolved electron densities from the natural orbitals {φ_*i*σ_^nat^} and their occupation numbers {*n*_*i*σ_^nat^} obtained from the MP2 calculations. These quantities are extracted from the relaxed MP2 one-particle density matrix using the implementation in the ricc2 module^62^ of TURBOMOLE within the analytic-gradient formalism.

12 $$\rho_{\sigma }=\sum_{i}n_{i\sigma }^{\mathrm{nat}}{\left|\varphi_{i\sigma }^{\mathrm{nat}}\right|}^{2},\sigma \in \left\{↑,↓\right\}$$

In the above equation, σ denotes the up and down spins. We then define the excess-electron density ρ_ex_ as the spin-density difference,

13 $$\rho_{\mathrm{ex}}=\rho_{↑}-\rho_{↓}$$

which provides a direct measure of the excess-electron density. ρ_ex_ is then compared with density ρ_b_ from the electronic bound-state wave function, as shown in the following equation:

14 $$\rho_{b}=|\psi_{b}^{*}{|}^{2}$$

We define the density difference (Δρ_ex–b_) using the following equation to assess how well the bound-state wave function ψ_b_^*^ reproduces the excess-electron density distribution.

15 $$\Delta \rho_{\mathrm{ex}-b}=\int {\left||\rho_{\mathrm{ex}}-\rho_{b}|\right|}_{2}$$

Furthermore, to ensure accurate density evaluation, we avoid grid-based real-space integration. Instead, we evaluate eq 15 directly from the Frobenius norm of the density matrix. All calculations are converged until the total-energy change is below 10^–7^ Ha and the root-mean-square (RMS) change in the electron density is below 10^–7^.

Our method relies on KS-DFT to describe solvent polarization and uses KS orbitals to construct the *G*_0_ *W*_0_ correction. The results, therefore, depend on the underlying exchange–correlation functional. In this study, we first present results obtained with CAM-B3LYP,^47^ which serves as the default starting point of our computational scheme. We then benchmark results obtained from other hybrid and range-separated functionals. In our test set, we consider the early global hybrid GGA functionals PBE0 ^44^ and B3LYP.^45,46^ PBE0 uses a nonempirical mixing scheme with 25% exact exchange,^44^ whereas B3LYP is semiempirical and includes 20% exact exchange. More recent developments have extended hybrid functionals into the meta-GGA regime with improved performance.^63–65^ We therefore also test the global hybrid meta-GGA functionals M06,^66^ which includes 27% exact exchange, and M06-HF,^67^ which includes 100% exact exchange. For further comparison, we consider Hartree–Fock (HF), which also includes 100% exact exchange but neglects electron correlation beyond the mean-field level.

Excess electrons are sensitive to self-interaction error, and global hybrid functionals only partially mitigate this problem. A more systematic, though still incomplete, approach is to use range-separated hybrids, which incorporate more exact exchange at long range.^63^ For example, our default CAM-B3LYP functional^47^ uses 19% short-range exact exchange and 65% long-range exact exchange. To test other range-separated hybrids, we also consider ωB97X,^68^ which contains 100% exact exchange in the long-range limit and approximately 16% exact exchange in the short range. We further consider LRC-ωPBE,^49,69^ which has zero short-range exact exchange. For LRC-ωPBE, we set the range-separation parameter (ω_LRC_) to 0.30 *a*_0_^–1^ as the starting point in our electronic bound-state calculations. Here, *a*_0_ is the Bohr radius.

A key feature of LRC-ωPBE is the range-separation parameter, denoted here as ω_LRC_, which controls the separation between short-range DFT exchange and long-range exact exchange. As demonstrated by Rohrdanz et al.,^49^ different values of the range-separation parameter can be optimal for different target properties, including atomization energies, barrier heights, electron affinities, and excitation energies. Similarly, for VDEs, we fit different values of ω_LRC_ in LRC-ωPBE to find the value that most closely reproduces the reference CCSD­(T) VDEs.

Our benchmark-based fitting procedure should be distinguished from Baer’s IP-tuning procedure, in which the range-separation parameter is determined by enforcing consistency between the negative highest occupied molecular orbital (HOMO) energy and the ΔSCF ionization energy.^33^ Baer’s IP-tuning procedure has the advantage of not relying on reference VDEs from higher-level theory or experiment. However, because it optimizes a different criterion, it may not reproduce the present CCSD­(T) VDEs as closely as the benchmark-based fitting procedure when high-accuracy VDEs are available. For CAM-B3LYP and ωB97X, the range-separation parameters were not fitted; these functionals were used with their standard parametrizations because their range-separation parameters are part of the original functional definitions rather than system-specific fitted parameters.^70^

All calculations were performed on a dual-socket workstation with two Intel Xeon E5-2690 v3 CPUs (24 physical cores in total; 2 × 12 cores at 2.60 GHz). Reported runtimes correspond to the wall-clock time, *T*_wall_, measured in seconds.

## 4 Results and Discussion

### 4.1 Test Set of Molecular Clusters and Benchmark Design

We select a diverse set of molecular clusters to represent complex solvent environments while keeping the system size computationally feasible. Because CCSD­(T), which scales as *O*(*N* ^7^), serves as the reference electronic-structure method throughout this study, the high-accuracy benchmarking of electronic bound states is restricted to clusters with *n* ≤ 5. Figure 1a shows representative water-cluster structures. Each structure is labeled by the number of monomers, the hydrogen-bond topology, and a two-letter code indicating whether the central water molecule acts as a proton donor (d) and/or acceptor (a). For example, in the 3Rda cluster, the water monomers form a ring, and each molecule both donates and accepts one proton. In contrast, in the 3Laa cluster, the central molecule accepts protons from its two neighboring monomers. For Y-type clusters, the two numbers indicate the monomers in the ring and chain, respectively.

**Figure 1:**
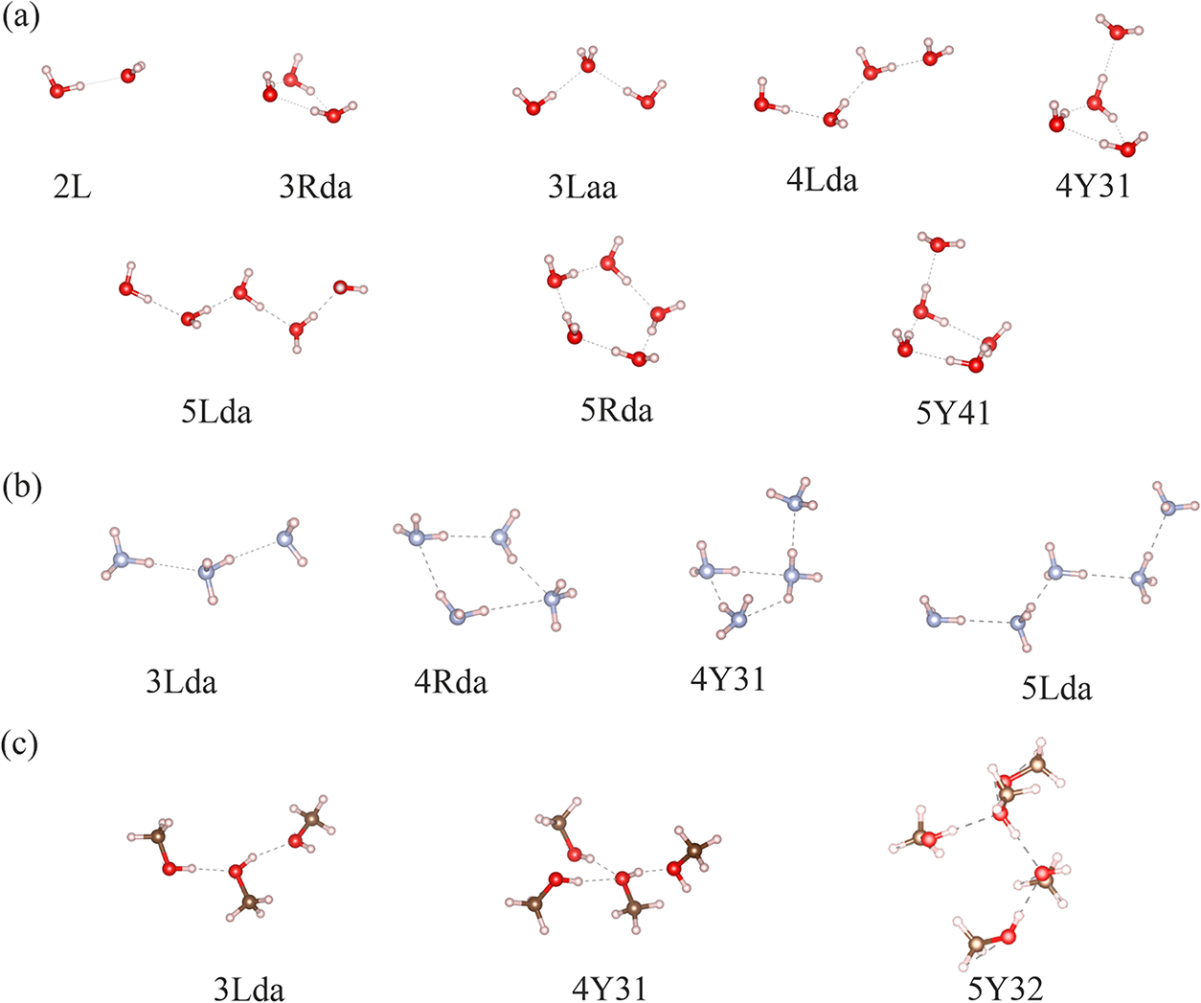
Overview of some selected anionic clusters considered in this study. Panels (a), (b), and (c) correspond to the water, ammonia, and methanol clusters, respectively.

For small anionic clusters, the excess electron is typically dipole-bound. Forming such states requires a preferential alignment of the monomer dipoles. For the linear (L) topologies in our set (2L, 3Laa, 4Lda, and 5Lda), the individual dipoles align predominantly along a common direction. For the ring topology 3Rda, the dangling O–H bond on each water monomer is oriented toward the same side of the ring plane, creating a net dipole moment perpendicular to the plane. The 4Y31 structure is derived from 3Rda by adding one additional water molecule on the same side of the ring plane. This extra monomer further increases the total dipole moment of the cluster.

Ammonia forms hydrogen-bonded networks similar to those of water. Accordingly, ammonia clusters exhibit the same topology patterns (Figure 1b), including L, R, and Y, and they also show a similar tendency toward parallel-dipole alignment. The ground-state dipole moment of an ammonia monomer is 1.51 D,^71^ which is smaller than that of a water monomer, 1.87 D.^72^ As a result, ammonia clusters of the same size tend to have smaller net dipole moments and support more weakly bound excess electron states. Consequently, at least three ammonia molecules are required to form a dipole-bound state,^73^ whereas a water dimer is sufficient to bind an excess electron.^28^

Methanol-cluster structures are more complex than those of water and ammonia because the methyl group does not participate in hydrogen bonding. As a result, methanol clusters tend to orient their O–H groups toward one another to form hydrogen bonds rather than align their dipoles in parallel. Consequently, methanol clusters typically support more weakly bound excess-electron states. In our test set, most methanol structures fall into the L or Y categories.

Given the variety of structures, clusters within the same topology exhibit similar dipole-moment arrangements and therefore have similar VDE values. Variations among structures within a topology lead to only small changes in the VDE (∼10 meV). Accordingly, we select only representative structures for each topology to avoid oversampling. In total, we consider 20, 14, and 14 clusters for water, ammonia, and methanol, respectively. All of the structures used in combination with CCSD­(T) benchmarking are available in the Supporting Information.

To assess the accuracy of the electronic bound-state energies and compare them with the commonly used MP2 and ΔSCF methods, we evaluate *E*_b_^*,XC^, −*E*_VDE_^MP2^, and −*E*_VDE_^XC^ directly against −*E*_VDE_^CCSD(T)^ for each cluster. Deviations from the CCSD­(T) reference are quantified using the mean absolute error (MAE) and the maximum absolute error (MaxAE). To characterize statistical trends across solvents, we also perform linear fits of these quantities against −*E*_VDE_^CCSD(T)^. The fitted slope *s* reflects the multiplicative bias relative to the CCSD­(T). When *s* ≠ 1, the absolute deviation increases approximately in proportion to |*E*_VDE_^CCSD(T)^|. We therefore define the percent slope deviation from unity as δ_*s*_ = |*s* – 1| × 100%. The fitted intercept *t* reflects a constant offset, or additive bias, across the VDE energy range, and its magnitude is quantified by |*t*|.

### 4.2 Benchmarking *E*_b_^*,XC^ against CCSD­(T) VDEs

To evaluate the accuracy of the electronic bound-state energies *E*_b_^*,XC^, we compare them with the VDEs calculated at the CCSD­(T) level of theory, *E*_VDE_^CCSD(T)^. In this subsection, we focus on the default starting point, XC = CAM-B3LYP. Figure 2a summarizes the MAE and MaxAE for the three solvents considered in this study. Water exhibits the largest absolute errors (MAE = 3.836 meV; MaxAE = 14.63 meV), followed by methanol (MAE = 3.820 meV; MaxAE = 6.301 meV). Ammonia shows the smallest errors (MAE = 1.381 meV; MaxAE = 2.439 meV).

**Figure 2:**
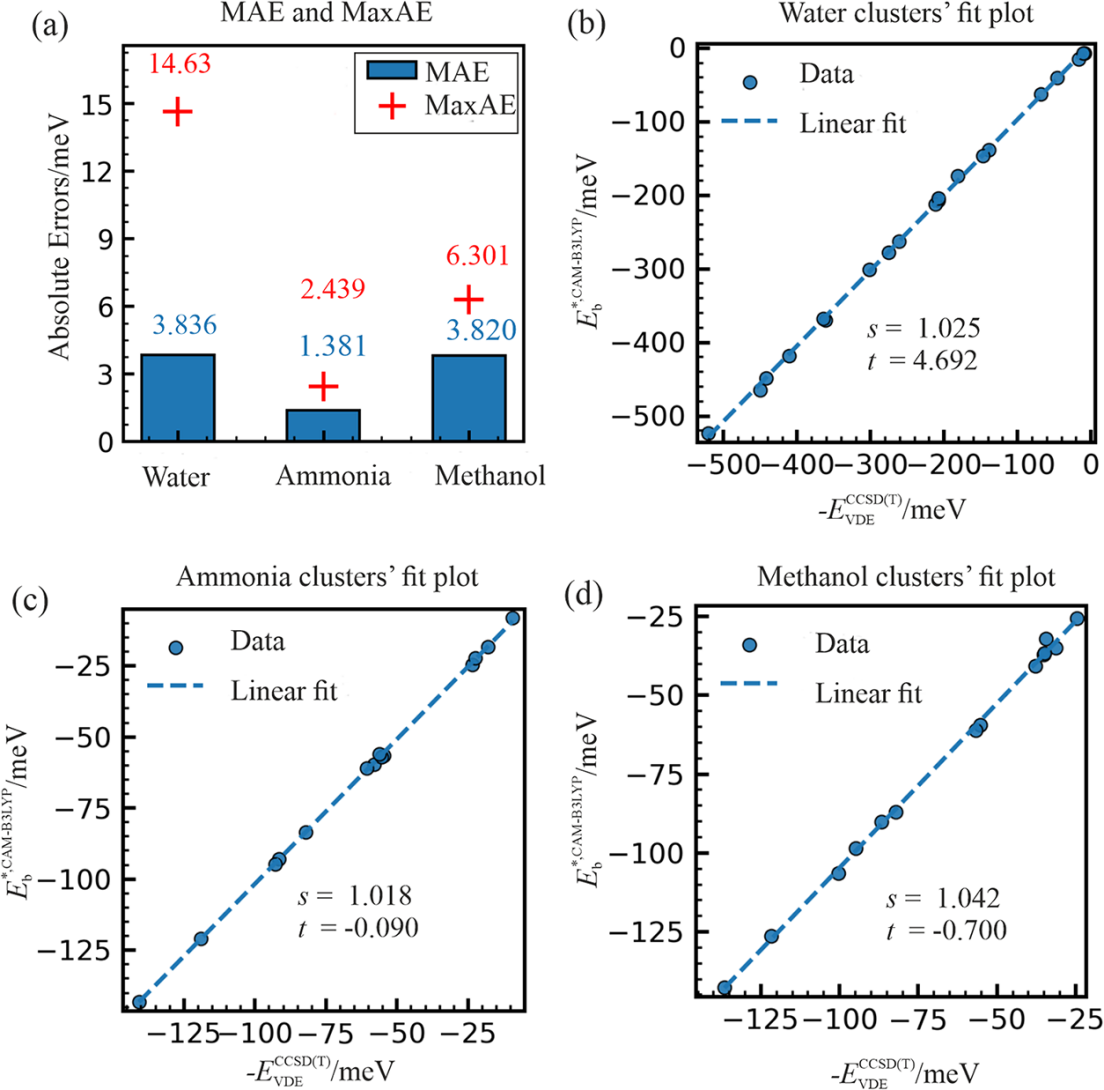
Comparison of *E*_b_^*,CAM‑B3LYP^ with −*E*_VDE_^CCSD(T)^. Panel (a) shows the MAE and MaxAE for the water, ammonia, and methanol clusters. The horizontal axis labels the solvent types, and the vertical axis shows the absolute errors between *E*_b_^*,CAM‑B3LYP^ and −*E*_VDE_^CCSD(T)^. The blue bars represent the MAE, and the red crosses denote the MaxAE. Panels (b)–(d) show the scatter plots and linear fits for the water, ammonia, and methanol clusters, respectively. Each scatter point corresponds to one cluster structure, and the dashed line denotes the first-order polynomial fit. The fitted slope and intercept are denoted by *s* and *t*, respectively. The horizontal axis is −*E*_VDE_^CCSD(T)^, and the vertical axis is *E*_b_^*,CAM‑B3LYP^; both are given in meV.

To understand the solvent-dependent MAE and MaxAE, we analyze how the error varies with the VDE within each solvent type using the fitted parameters. Figure 2b shows the scatter plot and fitting results for the water clusters, with δ_*s*_ = 2.5% and |*t*| = 4.692 meV. For water, the energy span Δ*E*_span_ of *E*_VDE_^CCSD(T)^ is approximately 520.0 meV. The maximum absolute error is therefore estimated as δ_*s*_ × Δ*E*_span_ + |*t*| = 17.69 meV, which is close to the observed MaxAE.

For the ammonia clusters, Figure 2c shows the smallest δ_*s*_ (1.8%) and |*t*| (0.090 meV). Consistent with these values, ammonia also exhibits the smallest MAE and MaxAE. By contrast, the methanol clusters in Figure 2d show the largest δ_*s*_ (4.2%). Although the Δ*E*_span_ of methanol (≈135.0 meV) is similar to that of ammonia, the MAE and MaxAE are significantly larger, primarily because of the larger δ_*s*_. However, because methanol has a much smaller Δ*E*_span_ than water, its MAE and MaxAE remain smaller than those of the water clusters. The linear fits for all three solvents also give coefficients of determination (*R* ^2^) greater than 0.99, showing that the electronic bound-state energies computed with our method are in close agreement with the CCSD­(T) VDEs. The data used to generate the scatter plots and the corresponding fitting results in Figure 2 are provided in the Supporting Information.

### 4.3 Starting-Point Dependence of *E*_b_^*,XC^ on the Exchange–Correlation Functional

Because the *G*_0_ *W*_0_ correction in our scheme is evaluated non-self-consistently, the computed *E*_b_^*,XC^ can depend on the exchange–correlation functional used to generate the initial KS orbitals and eigenvalues. We therefore assess the starting-point dependence of the method by benchmarking *E*_b_^*,XC^ obtained from different functionals against CCSD­(T) VDEs. This analysis allows us to evaluate the robustness of the proposed scheme with respect to the underlying functional choice and identify reliable starting points for the subsequent analysis.

The metrics used to evaluate the accuracy of different KS-DFT functionals are the same as those defined in Section 4.2, namely, MAE, MaxAE, δ_*s*_, and |*t*|. The *R* ^2^ values are omitted because all fits satisfy *R* ^2^ > 0.99. Figure 3a shows the MAE and MaxAE of *E*_b_^*,XC^ relative to −*E*_VDE_^CCSD(T)^ for different exchange–correlation-functional starting points in water, ammonia, and methanol. Among the range-separated functionals, CAM-B3LYP gives the smallest MAE and MaxAE, LRC-ωPBE gives the second-largest errors, and ωB97X gives the largest errors. Thus, the range-separated functionals show a consistent ordering of error magnitude across all three solvents.

**Figure 3:**
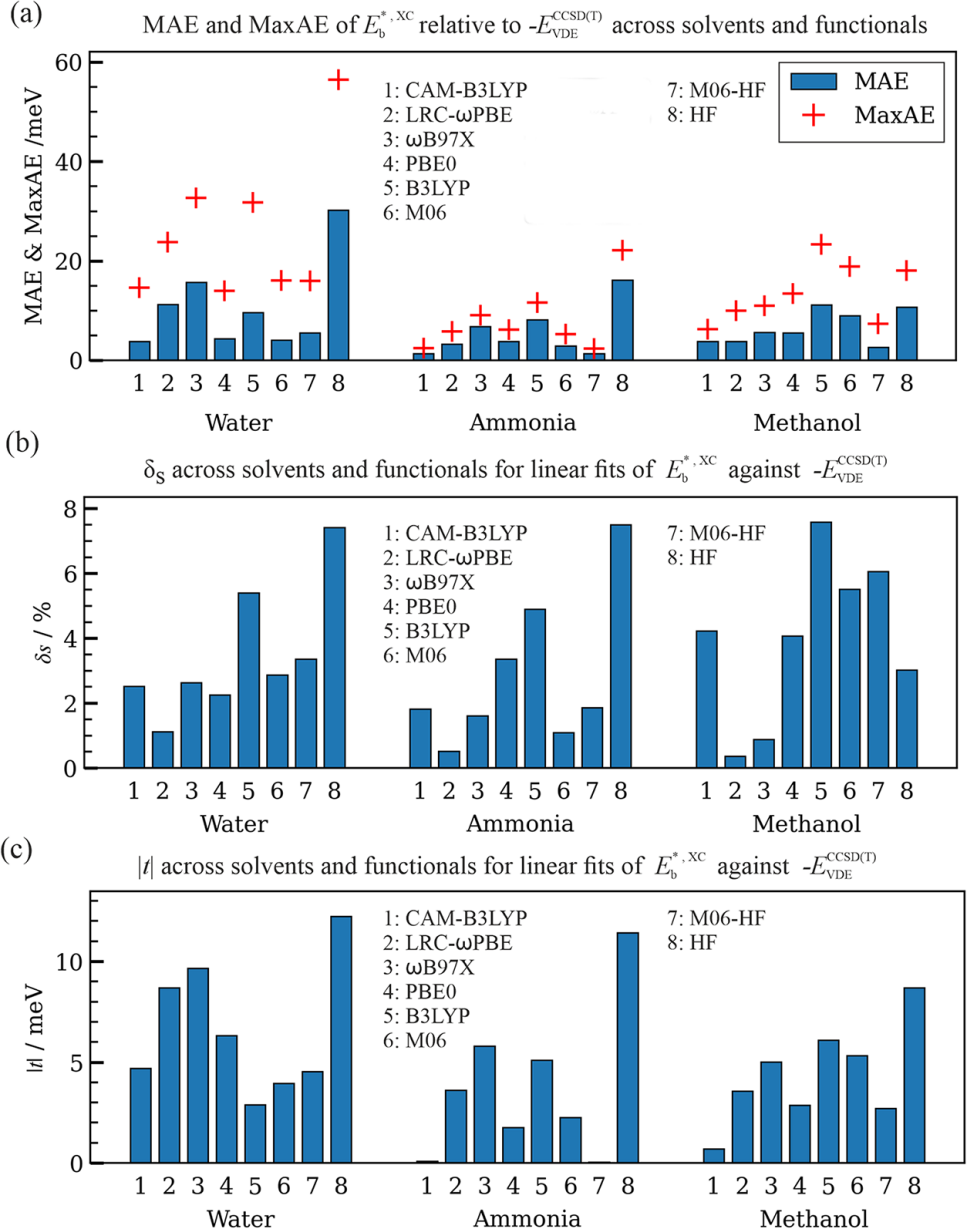
Comparison of *E*_b_^*,XC^ with −*E*_VDE_^CCSD(T)^ for different exchange–correlation-functional starting points. Panel (a) shows the MAE and MaxAE for water, ammonia, and methanol clusters. The horizontal axis groups the data by solvent, and the numbers denote the exchange–correlation functionals: CAM-B3LYP (1), LRC-ωPBE (2), ωB97X (3), PBE0 (4), B3LYP (5), M06 (6), M06-HF (7), and HF (8). The vertical axis is given in meV; red crosses indicate the MaxAE, and the bar heights represent the MAE. Panels (b) and (c) show δ_*s*_ and |*t*|, respectively, using the same horizontal axis as in panel (a). The vertical axis is given in % in panel (b) and in meV in panel (c).

Among the hybrid functionals, B3LYP gives the largest MAE and MaxAE values across all three solvents. For the other hybrid functionals, namely, PBE0, M06, and M06-HF, the errors depend on the solvent. In ammonia and methanol, M06-HF gives the smallest errors, whereas PBE0 performs the best for water. We also compare these results with those of HF, which neglects valence-electron correlation. HF gives the largest MAE and MaxAE for water and ammonia and the second-largest errors for methanol. These results show that including valence electron correlation through a KS exchange–correlation functional is essential for obtaining a reliable starting point.

For a quantitative comparison, the averaged MAE and MaxAE over all three solvents are 6.177 and 12.87 meV for the range-separated functionals, 5.708 and 13.86 meV for the hybrid functionals, and 19.04 and 32.24 meV for HF. Therefore, the range-separated and hybrid functionals have comparable overall errors, whereas HF gives the largest averaged MAE and MaxAE. The exact MAE and MaxAE values used in Figure 3a are listed in Table S1 of the Supporting Information.

The absolute errors directly quantify the small clusters considered in this study. However, to assess generality beyond the set of structures examined here, it is important to analyze δ_*s*_. A smaller δ_*s*_ indicates that the error grows more slowly with increasing VDE. Figure 3b shows that LRC-ωPBE has the smallest δ_*s*_ for all three solvents. Therefore, the LRC-ωPBE provides the most robust performance over a wider VDE range. In addition, other range-separated functionals, namely, CAM-B3LYP and ωB97X, have δ_*s*_ values that remain below 5.0% for all solvents.

For the hybrid functionals, B3LYP shows the largest δ_*s*_ across the different solvents, indicating larger errors for more strongly bound excess electrons. PBE0, M06, and M06-HF show similar trends for water and methanol, with PBE0 giving the smaller δ_*s*_. In ammonia, however, M06 and M06-HF give smaller δ_*s*_ values than PBE0. Overall, the averaged δ_*s*_ values across the three solvents are 1.7% for the range-separated functionals, 4.0% for the hybrid functionals, and 5.9% for HF. These results indicate that, on average, the range-separated functionals show smaller slope deviations than the hybrid functionals and HF. The exact δ_*s*_ values used in Figure 3b are available in Table S2 of the Supporting Information.

In contrast to δ_*s*_, which becomes more important at larger VDEs, |*t*| is particularly important for loosely bound electrons with small VDEs. For the range-separated functionals, Figure 3c shows that, in all three solvents, |*t*| increases in the order CAM-B3LYP < LRC-ωPBE < ωB97X. In contrast, the |*t*| values of the hybrid functionals depend strongly on the solvent, and no common trend is observed across all three solvents. Nevertheless, HF gives the largest |*t*| for every solvent, indicating that HF is not an ideal starting point, especially for loosely bound electrons. On average across the three solvents, the |*t*| values are 4.647 meV for the range-separated functionals, 3.654 meV for the hybrid functionals, and 10.79 meV for HF. The exact |*t*| values used in Figure 3c are listed in Table S2 of the Supporting Information.

In summary, the range-separated functionals provide the most consistent starting points for our calculations. First, the MAE, MaxAE, and δ_*s*_ metrics show systematic trends across different solvents. This suggests that their performance is more predictable across the solvent types considered. Second, the range-separated functionals have the smallest averaged δ_*s*_, indicating better transferability over a wider range of VDE values. Taken together, these results show that CAM-B3LYP, LRC-ωPBE, and ωB97X provide more robust starting points than the global hybrid functionals and HF for the solvents studied.

### 4.4 Comparison with VDEs from Range-Separated ΔSCF and MP2

Although ΔSCF calculations based on range-separated functionals have been used to investigate the VDEs of anionic clusters,^34,74^ highly accurate benchmark studies across different solvents remain rare. For small anionic clusters, reports of ΔSCF calculations with MAEs below 5 meV and MaxAE values below 15 meV have so far been uncommon. Nevertheless, we select three established range-separated functionals, namely, CAM-B3LYP, ωB97X, and LRC-ωPBE, and analyze the *E*_VDE_^XC^ values predicted by ΔSCF calculations. Compared with the CCSD­(T) VDE values, the MAEs exceed 95.40 meV and the MaxAE values exceed 145.0 meV for all three functionals. Thus, ΔSCF calculations based on established range-separated functionals yield large absolute errors in VDEs. The full set of ΔSCF error metrics is provided in Tables S3 and S4 of the Supporting Information.

In addition to using the default ω_LRC_ = 0.30, *a*_0_^–1^ of LRC-ωPBE, we fit the parameter ω_LRC_ to the reference VDEs from CCSD­(T). We first carry out a parameter scan in which ω_LRC_ is varied from 0.5, *a*_0_^–1^ to 10, *a*_0_^–1^ in steps of 0.5, *a*_0_^–1^. As ω_LRC_ increases, the MAE and MaxAE for water, ammonia, and methanol decrease asymptotically. The corresponding plots of MAE and MaxAE versus ω_LRC_ are shown in Figure 4. Therefore, for the ΔSCF VDE calculations, the best agreement with the CCSD­(T) benchmarks is obtained in the large-ω_LRC_ limit of LRC-ωPBE.

**Figure 4:**
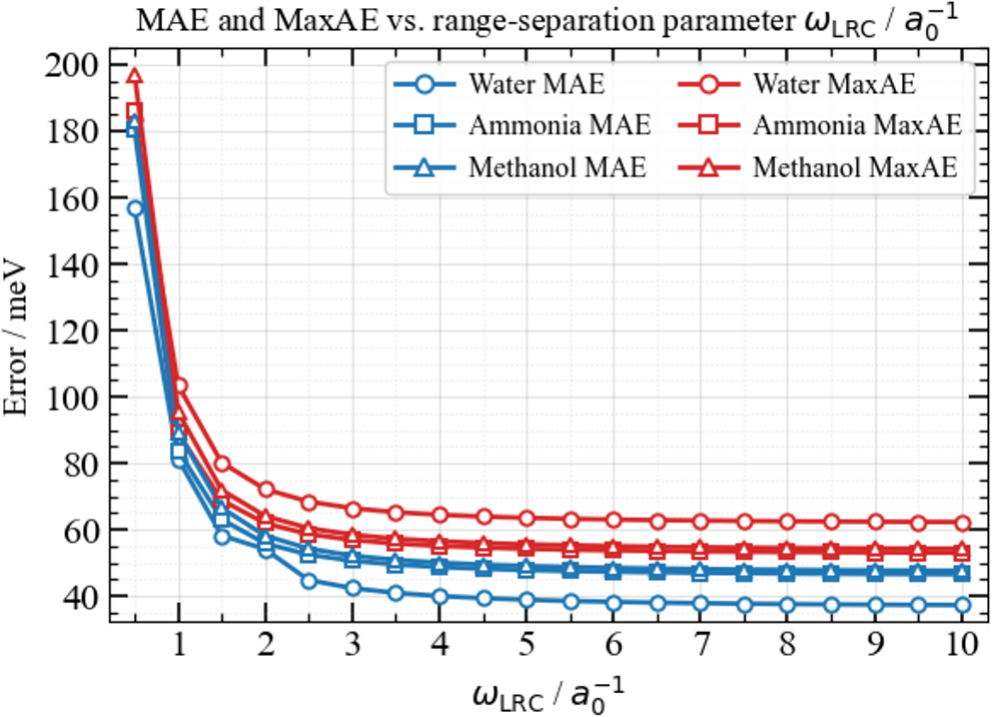
MAE and MaxAE of −*E*_VDE_^LRC‑ωPBE^ relative to the reference −*E*_VDE_^CCSD(T)^ versus the range-separation parameter ω_LRC_. The vertical axis shows the MAE and MaxAE in meV, and the horizontal axis shows ω_LRC_ in units of *a*_0_^–1^. Blue and red symbols denote the MAE and MaxAE, respectively. Circles, squares, and triangles correspond to water, ammonia, and methanol, respectively.

The benchmark-based fitting differs from Baer’s IP-tuning procedure. Baer’s IP-tuning procedure enforces consistency between the negative HOMO energy and the ΔSCF ionization energy rather than minimizing VDE errors with respect to CCSD­(T) benchmarks. Thus, the benchmark-based fitting procedure and Baer’s IP-tuning procedure need not lead to the same value of ω_LRC_. In the limit ω_LRC_ → ∞, the range separation assigns the full Coulomb interaction in the exchange part to the HF exact-exchange term. LRC-ωPBE, therefore, reduces to a functional containing full HF exchange and PBE correlation. The corresponding functional is denoted here as HF-PBE in the following discussion.

Table 1 summarizes the error metrics obtained by comparing −*E*_VDE_^HF‑PBE^ with the reference −*E*_VDE_^CCSD(T)^. These metrics include MAE, MaxAE, δ_*s*_, |*t*|, and *R* ^2^. For the water clusters, HF-PBE gives an MAE of 36.92 meV, which is close to the MAE of 35.2 meV reported by Yagi et al.^34^ Across the three solvents, the MaxAE ranges from 52.70 to 61.94 meV, similar to the range of |*t*| from 49.26 to 58.14 meV. Since the slope errors measured by δ_*s*_ are smaller than 10%, the constant offset |*t*| is the dominant contribution to the MaxAE.

**Table 1:** MAE, MaxAE, *δ*_*s*_, |*t*|, and *R* ^2^ for −*E*_VDE_^HF‑PBE^ Relative to −*E*_VDE_^CCSD(T)^ ^a^

metric	water	ammonia	methanol
MAE (meV)	36.92 (+33.08)	46.21 (+44.83)	47.37 (+43.56)
MaxAE (meV)	61.94 (+47.30)	52.70 (+50.26)	53.89 (+47.59)
δ_*s*_ (%)	9.168 (+6.646)	6.506 (+4.683)	2.738 (−1.485)
|*t*| (meV)	58.14 (+53.45)	50.32 (+50.23)	49.26 (+48.56)
*R* ^2^	0.9979 (−1.608 × 10^–3^)	0.9956 (−4.103 × 10^–3^)	0.9854 (−1.294 × 10^–2^)

a The columns correspond to the three solvents, water, ammonia, and methanol, and the rows list the different error metrics. MAE, MaxAE, and |*t*| are reported in meV, and δ_*s*_ in percent. For each metric, the values in parentheses give the difference relative to the corresponding value for *E*_b_^*,CAM‑B3LYP^ against −*E*_VDE_^CCSD(T)^.

To compare error metrics of −*E*_VDE_^HF‑PBE^ with *E*_b_^*,CAM‑B3LYP^, the values in parentheses in Table 1 show the differences relative to the corresponding error metrics discussed in Section 4.2. The error metrics obtained from ΔSCF calculations using the HF-PBE functional are systematically larger than those of *E*_b_^*,CAM‑B3LYP^, except for δ_*s*_ in methanol. The only decrease in δ_*s*_ for methanol is consistent with the analysis that the MaxAE arises primarily from the constant offset |*t*| rather than from the slope error. In addition, all *R* ^2^ values decrease for HF-PBE, indicating a slight deterioration in the fitting quality.

Since VDEs from range-separated functionals contain larger absolute errors relative to the CCSD­(T) reference values, we further analyze the VDEs computed at the MP2 level. Because MP2 and CCSD­(T) are both wave function-based *ab initio* methods, the corresponding MAE, MaxAE, and |*t*| values are systematically smaller than those from the ΔSCF calculations based on range-separated functionals. As shown in Table 2, the largest MaxAE of MP2 is 32.72 meV, which corresponds to the MaxAE for methanol. This maximum deviation arises from the 5Y32 methanol cluster, for which −*E*_VDE_^CCSD(T)^ and −*E*_VDE_^MP2^ are −100.2 and −67.46 meV, respectively. By contrast, the electronic bound-state energy of the same 5Y32 methanol structure, *E*_b_^*,CAM‑B3LYP^ = −106.5 meV, differs from the CCSD­(T) reference by only 6.3 meV. This example shows that, at least for some anionic cluster structures, *E*_b_^*,CAM‑B3LYP^ can provide a much more accurate estimate of the VDE than MP2.

**Table 2:** MAE, MaxAE, *δ*_*s*_, |*t*|, and *R* ^2^ for −*E*_VDE_^MP2^ Relative to −*E*_VDE_^CCSD(T)^ ^a^

metric	water	ammonia	methanol
MAE (meV)	19.65 (+15.81)	16.02 (+14.64)	17.40 (+13.59)
MaxAE (meV)	27.77 (+13.14)	21.74 (+19.30)	32.72 (+26.41)
δ_*s*_ (%)	3.315 (+0.7927)	4.919 (+3.095)	7.965 (+3.742)
|*t*| (meV)	11.98 (+7.286)	12.92 (+12.83)	12.11 (+11.41)
*R* ^2^	0.9995 (−8.500 × 10^–5^)	0.9958 (−3.876 × 10^–3^)	0.9828 (−1.554 × 10^–2^)

a The column, row, and values in the parentheses follow the same convention as in Table 1.

For a direct comparison of MP2 with *E*_b_^*,CAM‑B3LYP^, the values in parentheses in Table 2 show a systematic and substantial increase in all error metrics, including MAE, MaxAE, δ_*s*_, and |*t*|. Thus, across the three solvents considered here, MP2VDEs are overall less accurate than the present electronic bound-state energies. In particular, the δ_*s*_ value for methanol increases to 7.965%, indicating a larger slope error, especially for structures with larger VDE magnitudes. The coefficients of determination, *R* ^2^, also decrease systematically relative to those of the electronic bound-state energies, indicating that the quality of the linear fit is slightly worse for MP2. The underlying MP2 and CCSD­(T) VDE data are provided in the Supporting Information.

In summary, the electronic bound-state energies *E*_b_^*,CAM‑B3LYP^ closely reproduce the reference VDEs computed at the CCSD­(T) level of theory. Compared with the VDEs evaluated at the MP2 level, *E*_b_^*,CAM‑B3LYP^ shows systematically smaller errors, as demonstrated by the MAE, MaxAE, δ_*s*_, and |*t*| metrics. In addition, *E*_b_^*,CAM‑B3LYP^ is more accurate than the VDEs obtained from ΔSCF calculations using range-separated functionals. In particular, the MAE, MaxAE, and |*t*| metrics for *E*_b_^*,CAM‑B3LYP^ are systematically smaller than those for HF-PBE, which corresponds to the benchmark-fitted large-ω_LRC_ limit of LRC-ωPBE.

### 4.5 Spatial Distribution of the Electronic Bound-State Wave Function

In addition to energetic accuracy, we also examined the spatial distribution of the electronic bound-state wave function. Computational methods may yield accurate energies for different reasons; therefore, energetic agreement alone does not fully establish that the excess electron is described correctly. To address this concern, we compare the electronic bound-state wave function to the excess-electron density evaluated at the MP2 level of theory. If the wave function reproduces the spatial distribution of the excess electron well, then properties derived from the excess-electron distribution, including the center of mass (CM) and radius of gyration (RG), can also be determined accurately.

Nevertheless, the MP2 density matrix used for analytic gradients and response properties is an effective relaxed density and does not necessarily coincide with the exact one-particle reduced density matrix.^75^ We therefore assess the natural occupation numbers {*n*_*i*σ_^nat^} obtained from the ricc2 module of the TURBOMOLE package by monitoring violations of the physical bounds for spin–orbital occupations across all orbitals. In our calculations, the average violation is below 0.29% across all orbitals, and the maximum violation is 4.9%. For main-group compounds, violations of 5% or less are typically observed in the occupation numbers.^62,76^ Therefore, the MP2 natural occupations used here remain close to the physically allowed range and provide a practical approximation to the excess-electron density.

To assess whether the electronic bound-state wave function, ψ_b_^*,CAM‑B3LYP^, correctly reproduces the spatial distribution of the excess electron, we compare the density obtained from ψ_b_^*,CAM‑B3LYP^ with the excess-electron density computed at the MP2 level. The metric used to quantify this difference is Δρ_ex–b_, defined in eq 15. The averaged Δρ_ex–b_ values are 1.2 × 10^–11^, 5.4 × 10^–11^, and 4.2 × 10^–11^ for water, ammonia, and methanol clusters, respectively. These negligible Δρ_ex–b_ values indicate that ψ_b_^*,CAM‑B3LYP^ accurately captures the spatial distribution of the excess electron. Consequently, properties derived from the excess electron density, including the CM and RG, can also be reproduced accurately from ψ_b_^*,CAM‑B3LYP^ in our method.

Figure 5 shows examples of ψ_b_^*,CAM‑B3LYP^ and illustrates the localization of the loosely bound electron in different solvents. For each plot, we apply a uniform criterion to choose the isovalue such that the corresponding isosurface encloses 50% of the integrated excess-electron density.^77^ The resulting orbital plots allow a direct comparison of the spatial diffuseness of the excess electron.

**Figure 5:**
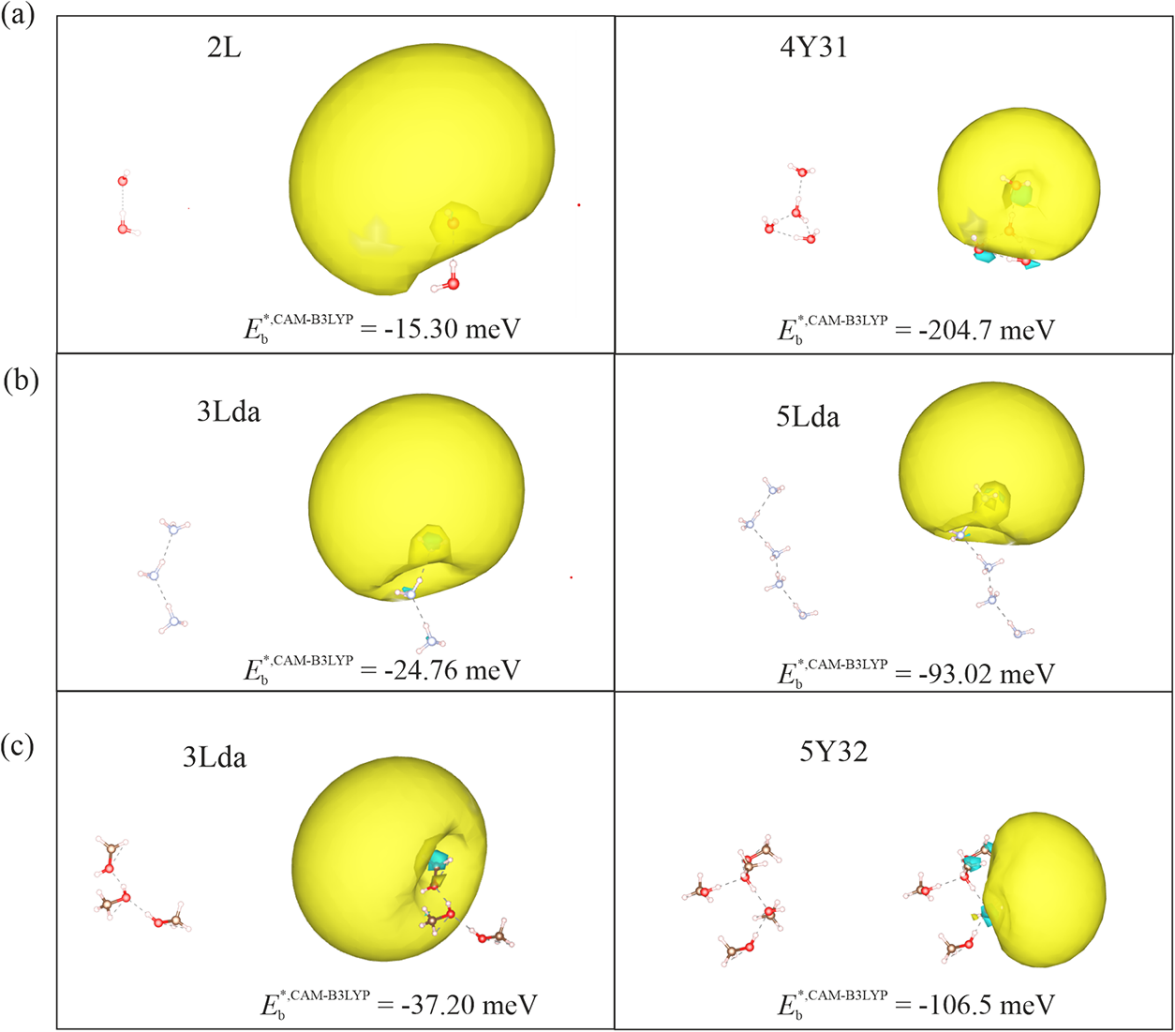
Isosurface plots of ψ_b_^*,CAM‑B3LYP^ for selected water (a), ammonia (b), and methanol (c) clusters. The cluster labels and corresponding electronic bound-state energies, *E*_b_^*,CAM‑B3LYP^, are indicated in each plot. To avoid obscuring the molecular structures with the isosurfaces, the cluster geometries are shown on the left side of each panel. The viewing angle and scale are identical to those used for the corresponding isosurface plots.

Figure 5a compares ψ_b_^*,CAM‑B3LYP^ for the 2L and 4Y31 water clusters. The corresponding values of *E*_b_^*,CAM‑B3LYP^ are −15.30 and −204.7 meV, respectively. The larger binding-energy magnitude of 4Y31 leads to a substantially more localized wave function. Figure 5b shows two representative ammonia clusters, 3Lda and 5Lda, with *E*_b_^*,CAM‑B3LYP^ values of −24.76 and −93.02 meV, respectively. Although 5Lda contains two additional monomers, the two binding energies remain within the same order of magnitude. Therefore, the corresponding electronic bound states exhibit similar diffusiveness. Figure 5c presents two exemplary methanol clusters, 3Lda and 5Y32, with *E*_b_^*,CAM‑B3LYP^ values of −37.20 and −106.5 meV, respectively. Consistent with the larger binding-energy magnitude, the wave function of 5Y32 is more localized than that of 3Lda.

From the excess electron distributions, we find that the spatial localization depends on both *E*_b_^*,CAM‑B3LYP^ and the solvent. For water clusters, *E*_b_^*,CAM‑B3LYP^ decreases more rapidly with an increasing cluster size, consistent with stronger localization of the excess electron. In contrast, for ammonia and methanol clusters, *E*_b_^*,CAM‑B3LYP^ decreases more slowly with the cluster size. As a result, water clusters of comparable size bind excess electrons more strongly, consistent with the larger VDEs of the aqueous electrons.

### 4.6 Scaling of the Computation Time

In this study, CCSD­(T), MP2, and ΔSCF are used for VDE calculations. Because of its rapidly increasing computational cost, CCSD­(T) is feasible only for clusters with size *n* ≤ 5 within our computational setup. In contrast, MP2 and ΔSCF can be applied to much larger clusters. To analyze the computational-time scaling, we therefore consider an additional set of larger molecular clusters with *n* ≤ 10 for all three solvents. For each cluster size *n*, we select only one structure from each solvent, namely, water, ammonia, and methanol, to avoid repeated sampling of structures of the same size. The set of structures used for the computational-time benchmark is available in the Supporting Information.

For each molecular cluster, the computational time of MP2 and ΔSCF is defined as the sum of the timings for the neutral and anionic calculations. For MP2, this total time includes both the initial HF calculation and the subsequent perturbative step. In practice, MP2 is commonly implemented with resolution-of-the-identity (RI) techniques,^78,79^ which are sufficiently efficient that the overall computation is often dominated by the initial HF step,^80^ with formal scaling of *O*(*N* ^3^).

Figure 6 shows the computational-time scaling of the electronic bound-state calculation, MP2, and ΔSCF. Over the range *N* ≤ 680, the electronic bound-state calculation is more expensive than MP2, which is also more expensive than ΔSCF. For a more detailed analysis of the computational cost, we divide the data into regions *N* < 340 and *N* ≥ 340 because the scaling behavior differs between them. For each segment of scattered data, we perform a first-order polynomial fit. The fitted slope corresponds to the effective scaling exponent *m* of the computational cost.

**Figure 6:**
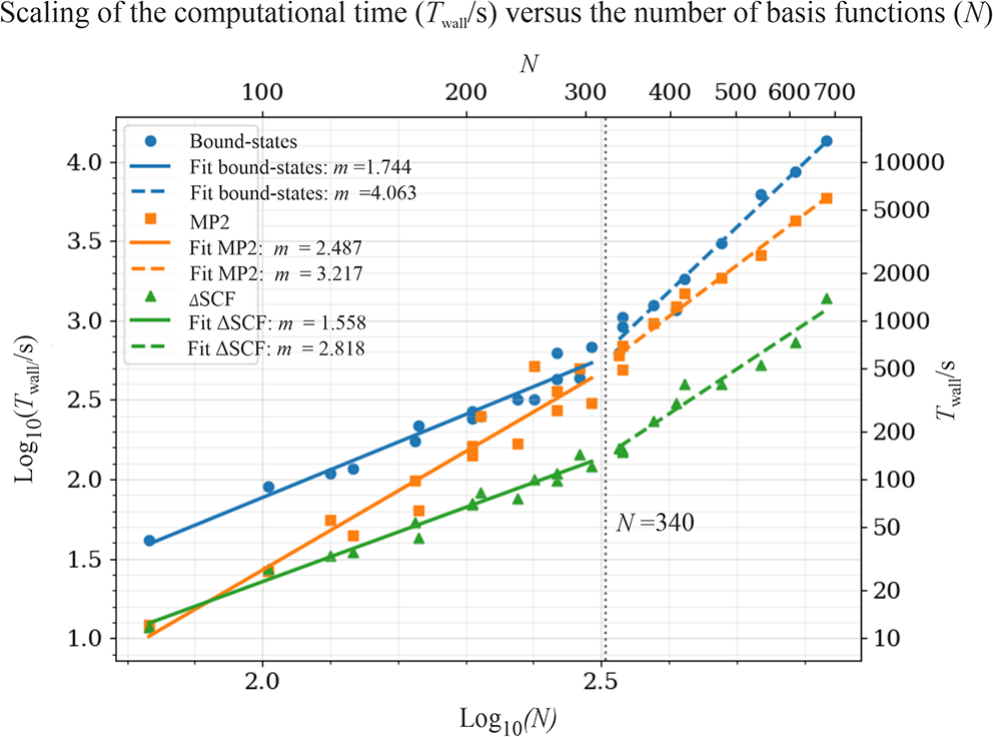
Time scaling of the electronic bound-state, MP2, and ΔSCF calculations. The left vertical axis shows the computational time as log_10_(*T*_wall_/s), and the right vertical axis shows the corresponding times in seconds. The lower horizontal axis shows log_10_(*N*), while the upper horizontal axis shows the corresponding number of basis functions, *N*. The vertical line at *N* = 340 separates the data into smaller- and larger-basis regimes. The blue circles, orange squares, and green triangles represent the computational times for the common set of structures evaluated using the electronic bound-state, MP2, and ΔSCF methods, respectively. The colors of the fitted curves follow the same convention as the scatter points. Solid lines denote the fits for the smaller-basis regime, whereas dashed lines denote the fits for the larger-basis regime. The slope of each fitted line is labeled as *m*.

For all line segments with *N* < 340, the electronic bound-state calculation, MP2, and ΔSCF show fitted *m* values of 1.733, 2.487, and 1.558, respectively. All values are smaller than the typical cubic scaling. This reduced scaling arises because, for small systems, the time-limiting step is the Fock build in the SCF procedure. In localized Gaussian basis sets, Schwarz screening removes most negligible two-electron integrals, so the practical cost of the Fock build can scale closer to *O*(*N* ^2^) with smaller basis functions.^81^

To examine the scaling in the larger-basis-function regime, we focus on structures with *N* ≥ 340. For the ΔSCF calculation, the fitted scaling is *m* = 2.818, which is close to the theoretical cubic scaling of 3.000. Therefore, the underlying SCF step begins to dominate in the *N* ≥ 340 regime. For MP2, the fitted scaling is *m* = 3.217, which is larger than 3.000 but still smaller than the formal *O*(*N* ^5^) scaling of the perturbative step. Consequently, for the structures considered here, the overall MP2 computational time is still dominated by the initial HF calculation, consistent with the efficient RI implementation. For the electronic bound-state calculation, the fitted scaling is *m* = 4.063, which is the largest among our benchmarks. This near-quartic scaling is consistent with conventional *G*_0_ *W*_0_ implementations, which typically scale as *O*(*N* ^4^).^82^

For a direct comparison, we use the largest structure considered here, (CH_3_OH)_10_^–^ with *N* = 680, as an example. The electronic bound-state calculation takes 13,480 s, whereas MP2 and ΔSCF require 5870 and 1388 s, respectively. Thus, at *N* = 680, the electronic bound-state calculation is 2.295 times more expensive than MP2, while MP2 is 4.229 times more expensive than ΔSCF. Averaged over all tested structures, the electronic bound-state calculation is 1.657 times more expensive than MP2, indicating that the two methods remain on a similar computational time scale. In contrast, MP2 is on average 3.130 times more expensive than ΔSCF, confirming that ΔSCF is the most computationally efficient method.

## 5 Conclusions and Outlook

We have presented a two-step hybrid computational scheme for electronic bound states of excess electrons. Using small anionic clusters as model systems, the resulting bound-state energies reproduce the reference VDEs computed at the CCSD­(T) level of theory. The fitted slope deviations remain below 5% across different binding strengths. In addition, the bound-state wave functions reproduce the spatial distribution of the excess electron. Together, these results show that our method provides a reliable description of both the energies and the spatial distribution of the excess electron. Furthermore, the close agreement of the electronic bound-state energies with the CCSD­(T) reference suggests that the key solvent–electron interactions are captured through the combined treatment of solvent polarization and electron–electron correlation.

In addition, the proposed computational scheme is robust with respect to the choice of starting point using different exchange–correlation functionals. In particular, the range-separated functionals, including CAM-B3LYP, LRC-ωPBE, and ωB97X, show consistently accurate reproduction of the VDEs across water, ammonia, and methanol clusters. Relative to the commonly used MP2 and ΔSCF methods, the electronic bound-state energies show systematic improvements in accuracy, as demonstrated by the MAE, MaxAE, |*t*|, and *R* ^2^ metrics. Notably, the MaxAE is at least 30 meV smaller than that of MP2 for the small anionic clusters considered here. These results indicate that our computational scheme provides a more accurate framework for studying excess electrons in different solvent clusters.

Finally, the computational cost of the electronic bound-state calculation is comparable to that of MP2 for molecular clusters containing up to 20 main-group atoms. For larger clusters, the computational cost shows quartic scaling, consistent with the *G*_0_ *W*_0_ step used in our approach to account for electron correlation effects. These timings show that the method provides a practical one-electron framework for excess electrons in small-molecular clusters. Future work can focus on further reduction of the computational cost. The approach can be extended to larger clusters to study the dynamics of the solvated electrons.

## Supplementary Material











## Notes

Tabulated linear-fit results and supporting data related to the analyses in Sections 4.3 and 4.4 (PDF)
Cartesian coordinates of all small anionic clusters benchmarked at the CCSD­(T) level of theory (ZIP)
Original data for the electronic bound-state energies and CCSD­(T) reference VDEs used to generate Figure 2 (XLSX)
Original MP2 and CCSD­(T) VDE data used to generate Table 2 (XLSX)
Cartesian coordinates of all small anionic clusters used in Section 4.6 for the computational-scaling benchmark (ZIP)
