# Supplementary material for: Computation of Electronic Bound States in Anionic Clusters as Precursors to Solvated Electrons
Source: J Chem Theory Comput. 2026 Jun 16;22(13):6635–47. doi: 10.1021/acs.jctc.6c00731 (PMC13374037; doi:10.1021/acs.jctc.6c00731)
Supplement: Supplementary file 1 [file ct6c00731_si_001.pdf]

Supporting Information: Computation of Electronic Bound States in Anionic Clusters as Precursors to Solvated Electrons

Xiangfei Wang*

Institute of Chemistry and Biochemistry, Freie Universität Berlin, Arnimallee 22, 14195 Berlin, Germany

E-mail: xfwangqc@zedat.fu-berlin.de

Table S1: MAE and MaxAE of $E_b^{*,\mathrm{XC}}$ relative to $-E_{\mathrm{VDE}}^{\mathrm{CCSD(T)}}$. Here, XC denotes the exchange-correlation functional used as the starting point of the calculation, including both range-separated and hybrid functionals, while HF is also included for comparison. The MAE and MaxAE are given in meV.

	Water		Ammonia		Methanol	
Functional	MAE	MaxAE	MAE	MaxAE	MAE	MaxAE
CAM-B3LYP	3.836	14.64	1.381	2.439	3.820	6.301
LRC-ωPBE	11.30	23.81	3.286	5.825	3.800	10.02
ωB97X	15.76	32.71	6.823	9.069	5.602	11.01
PBE0	4.397	13.95	3.868	6.201	5.579	13.49
B3LYP	9.675	31.79	8.183	11.60	11.15	23.31
M06	4.138	16.11	2.950	5.306	8.989	18.86
M06-HF	5.548	15.97	1.407	2.356	2.613	7.348
HF	30.27	56.47	16.16	22.15	10.70	18.10

Table S2: δ_s, $|t|$, and R^2 of the linear fits of $E_b^{*,\mathrm{XC}}$ against $-E_{\mathrm{VDE}}^{\mathrm{CCSD(T)}}$. Here, XC denotes the exchange-correlation functional used as the starting point of the calculation, including both range-separated and hybrid functionals. The HF is also included as a starting point for comparison. The values of $|t|$ are given in meV, δ_s in percent, and R^2 as the coefficient of determination.

	Water			Ammonia			Methanol		
Functional	δ_s	$\|t\|$	R^2	δ_s	$\|t\|$	R^2	δ_s	$\|t\|$	R^2
CAM-B3LYP	2.522	4.692	0.9995	1.823	0.090	0.9997	4.223	0.700	0.9983
LRC-ωPBE	1.124	8.695	0.9992	0.515	3.610	0.9992	0.361	3.560	0.9968
ωB97X	2.638	9.652	0.9991	1.608	5.809	0.9991	0.880	5.017	0.9969
PBE0	2.259	6.332	0.9994	3.356	1.752	0.9995	4.070	2.872	0.9945
B3LYP	5.399	2.885	0.9994	4.903	5.091	0.9994	7.593	6.104	0.9916
M06	2.873	3.945	0.9996	1.087	2.265	0.9993	5.513	5.323	0.9931
M06-HF	3.361	4.533	0.9996	1.860	0.056	0.9995	6.055	2.700	0.9971
HF	7.414	12.245	0.9993	7.505	11.428	0.9990	3.022	8.689	0.9958

Table S3: MAE and MaxAE of $-E_{\mathrm{VDE}}^{\mathrm{XC}}$ from ΔSCF calculations relative to $-E_{\mathrm{VDE}}^{\mathrm{CCSD(T)}}$. Here, XC denotes the different exchange-correlation functionals, including CAM-B3LYP, LRC-ωPBE, and ωB97X, used in the ΔSCF calculations. The benchmarks are carried out across water, ammonia, and methanol. The MAE and MaxAE are given in the unit of meV

	Water		Ammonia		Methanol	
Functional	MAE	MaxAE	MAE	MaxAE	MAE	MaxAE
CAM-B3LYP	201.5	236.1	181.3	190.1	178.2	191.3
LRC-ωPBE	246.2	281.0	309.1	320.4	309.6	328.9
ωB97X	95.40	145.0	153.8	163.2	154.4	165.4

Table S4: δ_s, $|t|$, and R^2 of the linear fits of $-E_{\mathrm{VDE}}^{\mathrm{XC}}$ from ΔSCF calculations against $-E_{\mathrm{VDE}}^{\mathrm{CCSD(T)}}$. Here, XC denotes the different exchange-correlation functionals, including CAM-B3LYP, LRC-ωPBE, and ωB97X, used in the ΔSCF calculations. The benchmarks are carried out across water, ammonia, and methanol. The values of $|t|$ are given in meV, and δ_s is given in percent. R^2 is the coefficient of determination.

	Water			Ammonia			Methanol		
Functional	δ_s	$\|t\|$	R^2	δ_s	$\|t\|$	R^2	δ_s	$\|t\|$	R^2
CAM-B3LYP	14.89	167.0	0.9953	4.920	178.2	0.9827	22.78	163.1	0.9759
LRC-ωPBE	4.920	257.6	0.9659	10.18	302.7	0.9774	23.14	294.2	0.8555
ωB97X	18.64	135.9	0.9612	8.470	159.1	0.9749	1.530	153.3	0.8986
